# Flow Cytometry-Based Rapid Assay for Antigen Specific Antibody Relative Affinity in SRBC-Immunized Mouse Models

**DOI:** 10.3390/ijms26083664

**Published:** 2025-04-12

**Authors:** Chunli Sun, Yuan Jiang, Shujun Liu, Qilin He, Chengyao Han, Dai Su, Hao Ma, Xingyu Guo, Yan Zhang, Fubin Li, Huihui Zhang

**Affiliations:** 1 Shanghai Institute of Immunology, Faculty of Basic Medicine, Key Laboratory of Cell Differentiation and Apoptosis of Chinese Ministry of Education, Shanghai Jiao Tong University School of Medicine, Shanghai 200025, China; sunchunligrace@sjtu.edu.cn (C.S.); jiangy-77@sjtu.edu.cn (Y.J.); lsj937175387@sjtu.edu.cn (S.L.); pjh348@sjtu.edu.cn (Q.H.); mr__stark@sjtu.edu.cn (C.H.); cafune73@sjtu.edu.cn (D.S.); mmahao@sjtu.edu.cn (H.M.); starguo@sjtu.edu.cn (X.G.); yanzhang09@sjtu.edu.cn (Y.Z.); 2 Center for Immune-Related Diseases at Shanghai Institute of Immunology, Ruijin Hospital, Shanghai Jiao Tong University School of Medicine, Shanghai 200025, China

**Keywords:** sheep red blood cells (SRBC), T cell-dependent antibody response (TDAR), antibody affinity maturation (AAM), immunomodulation, immunotoxicity testing, Src homology 2-containing inositol 5-phosphatase 1 (SHIP-1), flow cytometry

## Abstract

Sheep red blood cells (SRBC) has a long history as a classical T-cell dependent (TD) antigen. Due to its cost-effectiveness, easy accessibility, and ability to elicit a robust antibody immune response, SRBC continues to be widely used in studies related with humoral immunity modulation, vaccine development, and immunoactivity/immunotoxicity testing of bioactive agents. However, detecting the relative affinity levels of SRBC-specific antibodies in SRBC-immunized animal models remains challenging. Using flow cytometry, we established a detection system capable of quickly and accurately assessing the SRBC-specific antibody relative affinity levels in humoral samples (e.g., serum, tissue fluid) of SRBC-immunized mouse models. We further validated this method using affinity maturation-deficient mice, demonstrating that this method can distinguish affinity levels of the antibodies from different samples. This approach is simple and efficient, providing an accurate and effective technological solution for research on mechanisms of humoral immunity, antibody affinity maturation, vaccine response, and immunoactivity/immunotoxicity testing.

## 1. Introduction

The T cell-dependent antibody response (TDAR) is a key process in adaptive immune response to specific antigens. It involves interactions between T cells and B cells, ultimately leading to the production of high-affinity antibodies against specific antigens. This process plays a crucial role in anti-infection immunity, vaccination, and autoimmune diseases [1,2,3]. During TDAR, antibody affinity maturation (AAM) occurs mainly in the germinal centers (GCs) of secondary lymphoid organs [4,5]. Through a series of iterative processes involving somatic hypermutation and selection, B cells undergo affinity maturation to produce antibodies with increased binding affinity for the target antigen [6,7]. AAM plays a pivotal role in enhancing the effectiveness of the immune response. By generating antibodies with higher affinity for antigens, the immune system can more effectively neutralize and eliminate pathogens, while defective AAM leads to immunodeficiency [8,9]. AAM is thus also critical in vaccine development, as vaccines aim to induce a robust and long-lasting immune response. By understanding and harnessing the principles of affinity maturation, researchers can design vaccines that elicit strong and durable antibody responses against specific pathogens [10]. On the other hand, dysregulation of this process can contribute to the development of immunodeficiency or autoimmune diseases, highlighting the importance of maintaining proper regulation of AAM for immune homeostasis [11,12,13]. Moreover, TDAR with AAM as a pivotal feature is also an important indicator for the effects of immunomodulatory or immunologically toxic bioactive agents such as natural compounds from food, botanicals or microbial sources [14,15,16,17].

Sheep red blood cells (SRBC) is one of the most commonly used T-cell dependent (TD) model antigens for animal immunization in a variety of categories of studies ranging from immunobiological mechanisms of antibody response [18,19,20], monoclonal antibody development [21], and vaccine designs [22] to immunomodulation/immunotoxicity tests [23,24,25,26]. SRBC has a long history of such usage since the 1960s and is still actively and widely used nowadays, as has several advantages as a model TD antigen: SRBC immunization elicits a robust immune response, characterized by pronounced germinal center cell differentiation that can be easily detected. Additionally, SRBC is cost-effective and readily available due to its stable commercial supply, making it an economical choice for research purposes. Moreover, its simplicity in operation, as it does not require adjuvants, offers a convenient experimental setup. As a TD model antigen, SRBC immunization will induce the production of SRBC antigen-specific antibodies in the immunized animals, which can be detected by an enzyme-linked immunosorbent assay (ELISA) procedure [27]. Specificity of the produced serum antibodies against SRBC antigens were confirmed by these approaches.

The evaluation of the antibody response induced by SRBC includes readouts such as the extent of differentiation and proliferation of GC B cells, the level of serum SRBC-specific antibody titers post-immunization, and the affinity level of these SRBC-specific antibodies. The former two can indicate the magnitude/quantity of the immune response, while the affinity level can reflect the effectiveness/quality of this immune response. However, many studies have shown that the scale and quality of immune responses are not always consistent, depending on immunization/physiological conditions and individual genetic regulations [28,29,30]. Therefore, the affinity level of antigen-specific antibodies is an irreplaceable independent indicator for evaluating the quality/effective outcome of a TDAR. This makes the assessment of antibody affinity levels imperative in a study involving TDAR. However, despite the availability of relatively mature and commonly used methods for detecting SRBC-specific antibody titers [19,31,32], there is currently no convenient method for assessing the relative affinity levels of SRBC-specific antibodies.

Methods for estimating SRBC antiserum affinity reported previously have been indirect, with the readout on a cellular level rather than molecular level, and relies on plaque-forming assays or hemolysin transfer assays based on radioactive labeling of SRBC, which were time-consuming, labor-intensive, and may be potentially harmful to the health of the experimental operators [33,34,35]. These drawbacks limited their use, and nearly all studies involving SRBC-immunization could only conclude with titer determination. Therefore, we seek to develop a simplified, safe and efficient method for detecting the relative affinity levels of SRBC-specific antibodies. In this study, we have developed a method based on flow cytometry techniques to detect the affinity levels of serum SRBC-specific antibodies, with validation of the reliability of the method by monitoring the affinity increase according to the known time course of affinity maturation in a standard immunization procedure and by comparing relative affinity levels of serum from SRBC-immunized wild-type mice with that from the affinity maturation defective mouse models.

## 2. Results

### 2.1. Detection of the Binding of Serum SRBC-Specific Antibodies to SRBC

**Figure 1:**
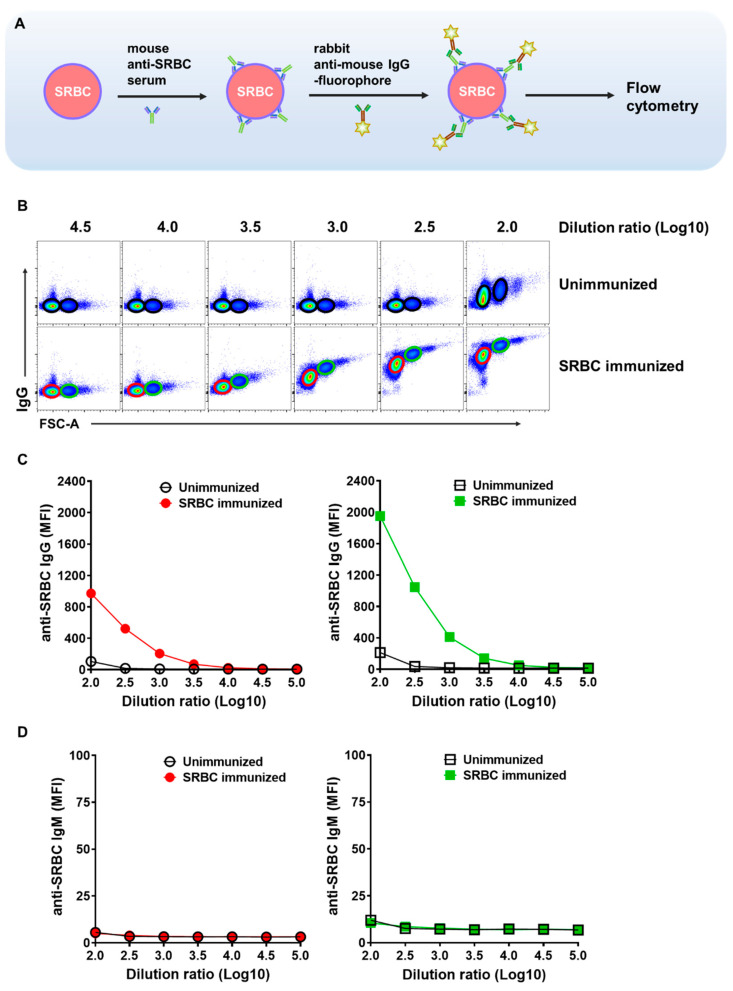
Detection of the binding of serum SRBC-specific antibodies to SRBC. (**A**) Schematic diagram of the detection of SRBC-specific antibodies binding to SRBCs using flow cytometry. (**B**) Flow cytometry plot of the binding of serial diluted serum from mice on day 14 of SRBC-immunization (lower panels) or from unimmunized mice (upper panels). In the lower panels, red circle gates indicate SRBC singlets and green circle gates indicate SRBC doublets. In the upper panels, black circle gates indicate SRBC singlets or doublets. (**C**) Plots showing the Mean Fluorescence Intensity (MFI) of the gated SRBCs of singlets (left panel) and doublets (right panel) in panel B. (**D**) Plots showing the MFI of gated SRBCs of singlets (left panel) and doublets (right panel) from incubation with the same set of serum samples as in panel B and C, albeit detecting them with fluorophore conjugated rabbit anti-mouse IgM secondary antibody.

The in vitro binding of antibodies in the serum of SRBC-immunized mice to SRBC was detected by flow cytometry. As shown in Figure 1A, SRBC were incubated with mouse anti-SRBC serum, letting the binding of SRBC-specific antibodies to the surface antigens of SRBCs. The SRBCs with bound antibodies were subjected to a further incubation with a fluorophore-conjugated rabbit anti-mouse IgG secondary antibody, which specifically binds to the SRBC-bound mouse IgGs. The fluorescence of the SRBCs was then detected by flow cytometry. Incubation of increasing amounts of serum from SRBC-immunized mice resulted in increasing binding signals, while the unimmunized mouse serum did not give an increasing signal (Figure 1B), validating the SRBC specificity of the assay system. The signal of SRBC doublets is about two-folds that of the singlets in each serum dilution ratio, showing that the experimental system is metrologically stable and reliable (Figure 1C). Since SRBC singlets constitute the main component of the detected cells, we used the signal of singlets as the readout in subsequent experiments.

To further confirm the specificity of the experimental system, we used another non-SRBC-cell line, the Jurkat cells, to conduct the above in vitro binding and flow cytometry experiments. This was performed to exclude the possibility of non-specific binding to non-SRBC-specific antigens on the cell surface. As shown in Figure S1, serum from SRBC-immunized mice produced a significant signal when incubated with SRBCs, whereas the same serum samples incubated with Jurkat cells exhibited a weak signal only near the lowest serum dilution, which was substantially much lower than the signal observed for SRBCs at corresponding dilutions. These results indicate that only at very low serum dilutions might there be minor non-specific binding, which has a negligible impact on the experimental system. Additionally, serum from unimmunized mice contained abundant IgG but showed almost no binding to either SRBCs or Jurkat cells (Figure S1), demonstrating that the signal generated by the binding of serum IgG from SRBC-immunized mice to SRBCs is specific and attributable to the presence of SRBC-specific IgG in these sera.

Another common form of non-specific binding is through the binding of Fc receptors on the cell surface. Fc receptors for IgG (FcγRs) are widely expressed by immune effector cells of hematopoietic origin, but are generally not found expressed by red blood cells [36,37]. To confirm the absence of such non-specific binding in this system, we pre-incubated SRBCs with an FcγR blocker prior to serum incubation. As shown in Figure S2, the titration curve of the Fc-blocked group and that of the untreated group are mostly overlapping, demonstrating that this experimental system is free from such non-specific binding.

We also used this flow cytometry (FC) system and the ELISA to determine the titer of a commercial rabbit anti-SRBC antibody, to further confirm the reliability of the system. As shown in Figure S3, both systems produced similar titration curves. Notably, the ELISA system exhibited a significant background signal, as shown by the non-specific binding signal of the serum of unimmunized mice, indicating that the FC system offers higher sensitivity and accuracy. This may be because the FC system only includes surface antigens of SRBC, resulting in higher specificity, while the ELISA system uses a full range of antigens including both surface and intracellular, which reduces the overall uniqueness of the antigens, leading to a certain degree of non-specific binding. Together, these results demonstrate that this flow cytometry system can specifically and accurately detect the binding of SRBC antibodies in test samples to SRBC.

Antigen binding of antigen specific IgM is usually not as efficient as IgG. Consistently, the signal generated by rabbit anti-mouse IgM secondary antibody of the same set of diluted serum from SRBC-immunized mice is significantly lower than that by anti-mouse IgG antibody and not distinguishable with serum from unimmunized mice in this experimental setting (Figure 1D). Moreover, class switched IgG rather than the unswitched IgM is a key feature of TDAR. Thus, we focused on IgG in the development of affinity assessing strategy.

### 2.2. Scheme of the Procedures for Assessing the Relative Affinity Levels of SRBC Antiserum

The fluorescence signal intensity generated by the binding of serum antibodies to SRBC was determined by both the quantity and affinity levels of SRBC-specific antibodies in the serum, both of which are unknown in a given sample. To mitigate the effect of differential SRBC-specific antibody quantity in different samples, we standardized the quantity of the bound antibodies to SRBCs by using the same starting binding signals in different samples. The affinity level was then able to be specifically detected by a subsequent procedure involving incubations of these samples with SRBCs under a serial of gradient elution conditions. By observing the trend of fluorescence signal reduction with increasing elution pressure across different samples, we could effectively assess the affinity levels.

**Figure 2:**
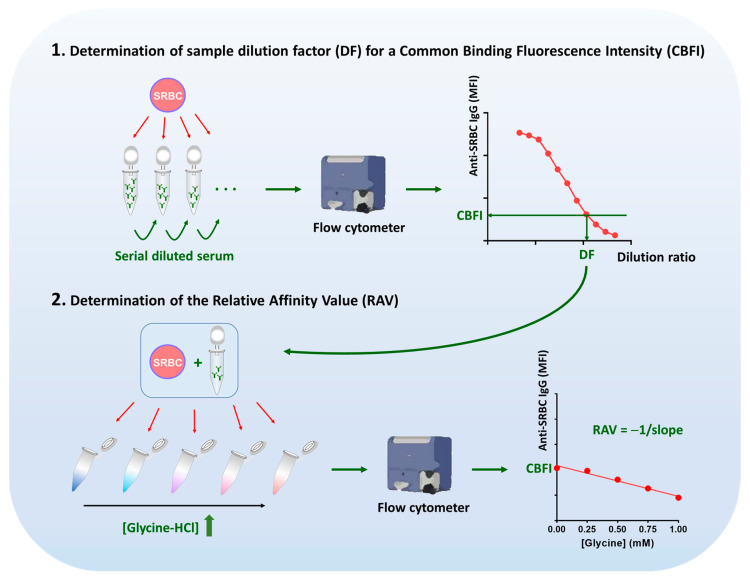
The scheme of the procedures for assessing the relative affinity levels of SRBC antiserum.

The specific experimental steps are illustrated in Figure 2. Initially, the samples were gradient-diluted, and each dilution was incubated with SRBC and subjected to flow cytometry to obtain a titration curve for the sample. All samples were tested for titration curves, and a signal intensity value falling within a linearly correlated range of dilution ratios and signal intensities across all samples was selected as a common binding signal fluorescence intensity (CBFI). Any value meeting this criterion was considered acceptable; however, the optimal CBFI was ideally situated in the middle portion of the linearly correlated range to avoid errors at the ends. It was determined that errors at the high signal end may result from saturation of SRBC antigens or result from selection of high affinity clones of low titer samples, while errors at the low signal end may result from a signal that is too low and causing a small reading window. Upon an appropriate CBFI was selected, the respective corresponding dilution factor (DF) values for each sample was determined by reading the dilution at which this CBFI was reached on the respective curves of each sample. In the linearly correlated range of dilution and fluorescence signal intensity, where a CBFI was selected, samples reaching the same CBFI value indicated an equal number of antibodies binding to the SRBC.

**Figure 3:**
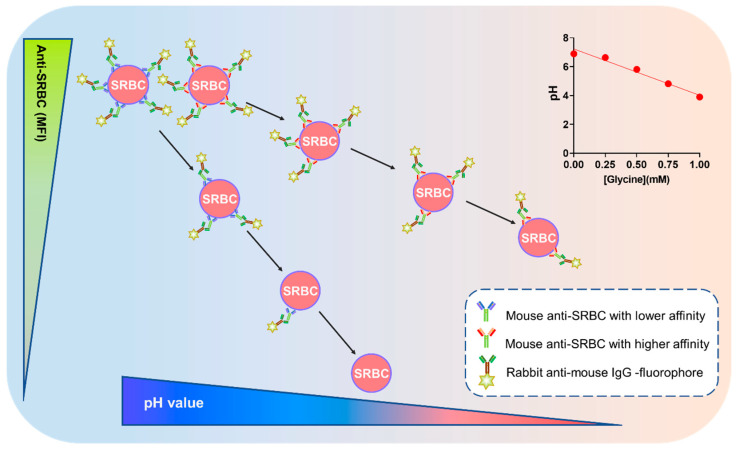
The principle of FCRA. (Internal upper right panel) pH values of the serial incubation solutions detected using a pH meter.

Normal saline was mixed with Glycine-HCl elution buffer (0.1 M, pH 2.7) in varying proportions to prepare a series of incubation solutions with gradually increasing acidity (Figure 3, Internal upper right panel), leading to a gradual increase in elution pressure. This was performed to create an environment in which the solution gradually becomes less favorable for binding of antibodies in the samples to SRBCs.

Samples diluted by the DFs corresponding to the selected CBFI were separately incubated with SRBCs in this series of incubation solutions with increasing acidity and elution pressure, labeled with fluorophore-conjugated secondary antibody and subjected to flow cytometry (Figure 2). It can be inferred that samples with higher antibody affinities would exhibited slower signal reduction with increasing acidity, reflected by smaller absolute values of the slope of the linear fit line. Conversely, samples with lower antibody affinities would show faster signal reduction with increasing acidity, indicated by larger absolute values of the slope of the linear fit line (Figure 3). The negative reciprocal of the slope was used as a measure of relative affinity level, termed the Relative Affinity Value (RAV), as shown by Equation (1):

RAV = −1/slope.(1)

This assay for assessing the relative affinity levels of SRBC antiserum is termed the Flow Cytometry based Relative affinity Assay (FCRA).

### 2.3. The Post-Immunizational Progressively Increased Relative Affinity Levels of SRBC-Immunized Wild-Type Mice Antisera Can Be Detected with FCRA

**Figure 4:**
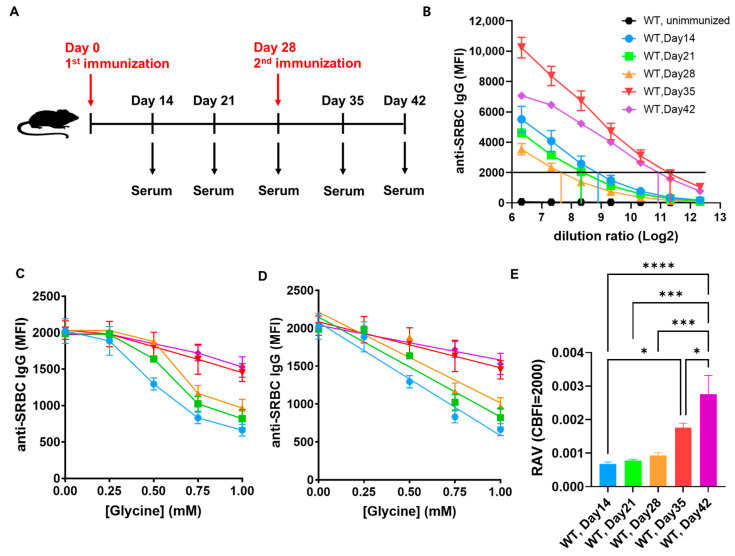
The relative affinity levels of SRBC-immunized wild-type mice antisera progressively increases after the initiation of immunization. (**A**) Schedule of mouse SRBC immunization and sample collection. (**B**) Titration curves of sera collected at the indicated time points (*n* = 5). (**C**) FCRA affinity detection curves (with CBFI as 2000) of the samples in panel B. (**D**) Linear fit line plots of the data in panel (**C**). (**E**) Relative affinity level values (RAV) calculated from the lines in panel (**D**). Data are represented as mean ± SEM. Ordinary one-way ANOVA was used in (**E**). * *p* < 0.05, *** *p* < 0.001, **** *p* < 0.0001.

It is known that, upon immunization of animals with an antigen, the affinity of the antigen specific antibodies produced will gradually increase over time [38]. Based on this, we applied an immunization schedule in C57BL/6J wild-type (WT) mice, as depicted in Figure 4A, where the first immunization happened on day 0, the second on day 28, and serum samples were collected weekly, from the second week post-immunization (day 14) to the sixth week post the first immunization (second week post the second immunization, totaling day 42). Using FCRA, we assessed the affinity of SRBC-specific antibodies in these sera. Figure 4B shows their titration curves, illustrating titers at days 28, 21, 14, 42, and 35 from low to high. The DF values corresponding to a CBFI of 2000 for each sample were obtained from the titration curves. By standardizing the starting point of detection to the CBFI of 2000 using these DF values, the affinity of each sample was tested. As shown in Figure 4C, the affinity levels at days 14, 21, 28, 35, and 42 gradually increases from low to high. Linear fitting of the data from Figure 4C yielded Figure 4D; the RAV for each sample were calculated using the formula mentioned in the previous section, as shown in Figure 4E, indicating a significant increase in affinity levels with immunization time, notably after the second immunization, consistent with previously reported results [38]. These results support the reliability and accuracy of FCRA in detecting the relative affinity levels of SRBC-specific antibodies in serum samples.

### 2.4. The AAM-Deficient ShipΔB Mice Antisera Has Lower Affinity Compared to That of WT Mice

**Figure 5:**
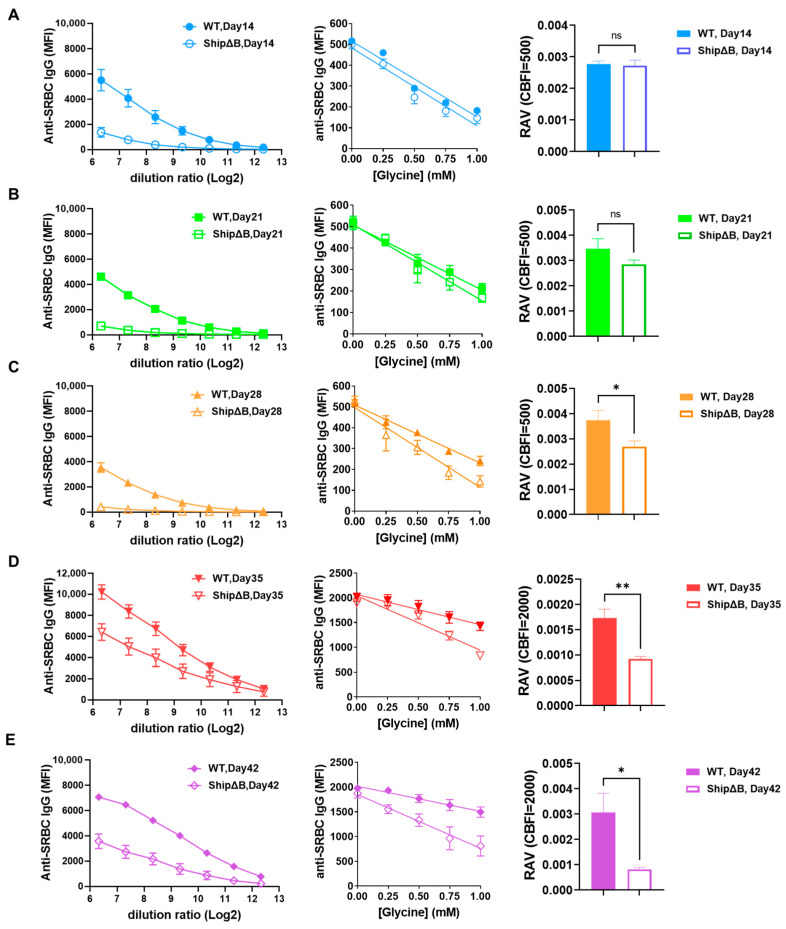
The AAM-deficient ShipΔB mice antisera have lower affinity compared to that of WT mice. (**A**–**E**) represent the FCRA results of serum samples on days 14, 21, 28, 35, and 42 of SRBC immunization, respectively. The left, middle, and right of each panel show the titration curves, linear fit line plots of affinity detection data, and RAV for samples at the corresponding time points, respectively (WT: *n* = 5, ShipΔB: *n* = 3). The data are represented as mean ± SEM. An unpaired *t*-test was used. * *p* < 0.05, ** *p* < 0.01, ns: non-significant.

In our previous work, we identified the B lymphocyte-specific Src homology 2-containing inositol 5-phosphatase 1 (SHIP-1)-deficient mice (ShipΔB) as an AAM-deficient model, which is unable to produce high affinity antibodies [39]. We used FCRA to assess the affinity levels of post SRBC immunization sera in this mouse model. Due to the very low titers of antibodies generated after the first immunization in ShipΔB mice, the signal value of 2000 is in the saturation range rather than the linear range of the correlation between dilution folds and fluorescence signals in these samples. Therefore, we used a CBFI value of 500 for the testing of all first immunization serum samples. After the second immunization, ShipΔB mice produced antibodies with higher titers, so we continued using a CBFI value of 2000 for testing all second immunization serum samples. As shown in Figure 5, under the same immunization protocol, the AAM-deficient ShipΔB mice failed to produce antibodies with increasing affinity over time like the wild-type mice. Starting from day 28, the affinity of SRBC antibodies in the serum of ShipΔB mice was significantly lower than that in the serum of wild-type mice at the correspondingly same time points. These results further validate the reliability and accuracy of the FCRA method.

## 3. Discussion

In this work, we developed a new experimental method, i.e., FCRA, to rapidly, accurately, and conveniently detect the relative affinity levels of post-SRBC immunization mouse sera. Taking advantage of the capability of flow cytometry to identify and analyze individual cells, we utilized intact SRBC naturally expressing antigens on their surface to detect the binding number of SRBC-specific antibodies in the serum sample of SRBC-immunized animals. By setting a CBFI, we standardized the starting bound number of SRBC-specific antibodies among different samples. This approach eliminates the influence of the quantity of specific antibodies in the samples, enabling a specific assessment of the affinity levels of these antibodies. We then developed conditions for measuring the affinity levels: using a series of incubation solutions with gradually decreasing pH values. In these incubation solutions, each sample starts at the same CBFI point, and the slope of signal changes with the acidity of the incubation solution differs based on the different affinities of the samples, allowing for the comparison of relative affinity levels among different samples.

In comparison to previously reported methods for detecting SRBC-specific antibody affinity levels [33,34], FCRA offers several advantages. Firstly, it is more convenient and efficient. The operational analysis time for one experiment is approximately 2 h per sample, and for multiple samples, the use of multi-well plates for simultaneous incubation and multi-channel pipettes for high-throughput operations can achieve an efficiency of approximately 5 h for 10 samples. Additionally, this method is cost-effective. SRBCs are commercially available and stable, with prices much lower than other protein-based commercial antigens. Using SRBCs as cells expressing natural antigens eliminates costs associated with antigen preparation and fixation on detection media, e.g., plates or beads. Lastly, we validated the accuracy of this detection method by using samples with known differences in affinity levels. The results show that comparing relative affinity data between samples using FCRA is reliable and accurate. Moreover, our results confirm the notion that titer levels and affinity levels do not always completely align, such as the titer levels of IgG antibodies peaking at days around 14 during the first immunization, followed by a decrease, and peaking at around day 7 (35 from the first immunization) during the second immunization, followed by a decrease as well. However, the affinity levels consistently increase from day 14 to day 42. This difference in the changing profile between titer and affinity is consistent with the results in previous findings [38].

Although the method of detecting the titers of SRBC-specific antibodies using enzyme-linked immunosorbent assay (ELISA) is well-established [31], there is not yet any report for detecting the affinity of these antibodies on an ELISA platform. The preparation of SRBC membrane antigens in ELISA protocols is complex and time-consuming, and commercial SRBC antibody ELISA test kits are not designed for measuring antibody affinity and are costly. Moreover, the strength of antigen binding to the ELISA plate may not withstand the elution conditions required for affinity measurements, which is why we did not explore the design and experimental conditions for SRBC antibody affinity detection on the ELISA platform.

Using the 4-hydroxy-3-nitrophenyl acetyl (NP) hepten conjugates with protein antigens (such as NP-CGG) for immunizing mice allows the evaluation of relative affinity levels based on the differences in binding capacity of mouse serum samples with different valency NP conjugates (such as NP3/NP23) [40,41]. However, for studies using SRBCs as antigens, switching antigens and immunizing experimental animals with other antigens would entail additional work and costs (will need additional experimental materials and animals). We compared the sensitivity of FCRA with the method using NP as the antigen for affinity detection. In the study by Cumpelik et al., there was no difference in affinity (np2/np24 ratio) between AAM-deficient mice and wild-type mice at the first immunization on day 12 [40]. In the study by Ye et al., there was no difference in affinity (np3/np23 ratio) between bcl6 null mice and wild-type mice at the first immunization on day 5, with significant differences only observed starting on day 5 of the second immunization [41]. These findings align with the results observed in our study with SRBC as antigen, where there was no significant difference in affinity between AAM-deficient mice (ShipΔB) and WT mice at the first immunization on day 14, with significant differences emerging on day 28 and after the second immunization (Figure 5). Thus, the sensitivity of FCRA and that of the method using NP as the antigen for affinity detection is comparable.

While the FCRA developed in this study demonstrates strong utility for rapid affinity comparison, it should be noted that this methodology specifically measures relative affinity levels of SRBC antiserum rather than absolute dissociation constants (kD) in standardized concentration units. This inherent limitation stems from two fundamental characteristics of the experimental system: the natural antigenic diversity present on SRBC surfaces and the polyclonal nature of serum antibodies with undefined concentrations of antigen-specific immunoglobulins. However, these constraints align appropriately with the primary requirements of humoral immunity research—the principal application domain of SRBC immunization models. These studies typically prioritize comparative assessments of immune potency across experimental groups over precise quantification of absolute affinity values. The FCRA directly addresses this predominant need by enabling efficient parallel evaluation of relative affinity profiles between biological samples. This capability suffices for most experimental scenarios of related study. Should absolute affinity determination become essential for specific investigations, such requirements could be addressed through supplemental methodologies including (but not limited to) SRBC membrane antigen purification and serum antibody purification protocols. Nevertheless, the current FCRA system remains optimized for the majority of applications where relative affinity ranking provides sufficient experimental resolution.

To conclude, FCRA provides a solution that researchers can rapidly, conveniently, and accurately detect the relative affinity levels of the produced specific antibodies of a TD response induced by SRBC immunization, thus may support research on humoral immunity related topics, such as the antibody affinity maturation, vaccine response, and immunoactivity/immunotoxicity testing of bioactive agents.

## 4. Materials and Methods

### 4.1. Animals

ShipΔB mice were kindly provided by Professor Jeffrey Ravetch at Rockefeller University in New York, USA. C57BL/6 wild-type mice (2–3 months of age) were purchased from Lingchang Biotechnology Co., Ltd, Shanghai, China. All animals were kept at the animal facility of Shanghai Jiaotong University School of Medicine (SJTUSM) Department of Laboratory Animal Science in specific pathogen-free (SPF) conditions, which maintains the temperature at 20–26 °C, the relative humidity at approximately 30–70%, a 12 h light cycle with living in individual ventilated cages (IVC), and with experiments conducted using age- and sex-matched mice to ensure experimental consistency. All experiments were approved and conducted in compliance with institutional guidelines and had been approved by the SJTUSM Institutional Animal Care and Use Committee (IACUC).

### 4.2. SRBC Immunization of the Mouse Models and Antiserum Preparation

An intraperitoneal injection of SRBC-saline solution containing 1 × 10^9^ SRBC per mouse was administered as a single immunization. Prior to injection, the cell concentration of the commercially available 4% SRBC solution (SenBeiJia Biological Technology Co., Ltd., Nanjing, China, Cat#SBJ-RBC-S003) was verified by diluting 1000-fold and counting under a microscope. The required numbers of SRBC were then aliquoted into a 15 mL centrifuge tube, centrifuged at 600× *g* for 5 min at 4 °C to remove the supernatant Alsever’s storage solution, and washed with 10 mL Phosphate-Buffered Saline (PBS) pH7.0 (HyClone, South Logan, UT, USA, Cat#SH30256) by the 600× *g*, 5 min centrifugation before resuspension to an appropriate concentration for *i.p.* injection. Following immunization, blood was collected from the mice weekly, and the serum was separated by centrifugation of the coagulated blood at 9000× *g* for 10 min and collecting the clear supernatant, then stored at −20 °C.

### 4.3. Determination of the DF Values of the Test Samples for a CBFI

The total required amount of SRBC, following the standard of 2 × 10^7^ SRBCs per well of a 96-well plate, was added into a centrifuge tube, centrifuged at 600× *g* for 5 min at 4 °C, and the supernatant was removed, followed by washing with PBS for twice before resuspended to an appropriate concentration in PBS. The cells were then added to a 96-well U-bottom plate with 2 × 10^7^ SRBCs in 100 μL per well. The serum samples were diluted in a 2-fold gradient series starting from 1:80, added to the 96-well plate planted with SRBC, mixed well, and incubated at 4 °C for 20 min. All wells of SRBCs were then washed twice with PBS by the above-mentioned centrifugation procedure before resuspended to 100 μL FACS buffer (PBS containing 1 mM ethylene diamine tetra acetic acid (EDTA), 2% Fetal Bovine Serum (FBS) (ExCell Bio, Shanghai, China, Cat#FSP500) with 1 μg/mL Alexa Fluor ^TM^ 488 rabbit anti-mouse IgG (H + L) antibody (Invitrogen, Carlsbad, CA, USA, Cat#A-11059) or Alexa Fluor ^TM^ 647 rabbit anti-mouse IgM antibody (Bioss, Beijing, China, Cat#bs-0368R-AF647) and incubated at 4 °C in the dark for 20 min. Following incubation, the SRBCs were washed with PBS by the above-mentioned centrifugation procedure twice before resuspended in FACS buffer for 200 μL per well, and the detection was performed using a flow cytometer (LSRFortessa TM X-20, BD, Franklin Lakes, NJ, USA), generating the titration curves of the test samples.

All samples were tested for titration curves, and a signal intensity value falling within a linearly correlated range of dilution factors and signal intensities across all samples was selected as CBFI. The respective DF values for each sample corresponding to the selected CBFI were then obtained from the titration curves of the samples.

### 4.4. Preparation of the Serial Glycine-HCl Incubation Solutions

The glycine-HCl serial incubation solutions were prepared by mixing normal saline (0.9% NaCl) with glycine-HCl elution solution (0.1 M, pH2.7) at ratios of 399:1, 199:1, 132:1, and 99:1 by volume to obtain glycine concentrations of 0.25 mM, 0.5 mM, 0.75 mM, and 0.1 mM with gradually decreasing pH values of the glycine-HCl incubation solutions (Figure 3, Internal upper right panel). Chemicals (Sodium chloride and Glycine) were purchased from Sangon Biotech Co., Ltd, Shanghai, China, and were of analytical grade.

### 4.5. Determination of SRBC Antiserum Relative Affinity Level with Serial Pseudo Eluting Incubation Starting from a Pre-Defined CBFI

The serum samples were diluted according to the DFs corresponding to the selected CBFI value and mixed with the prepared different gradient levels of glycine-HCl sample incubation solutions for 100 μL per well. The mixtures were then transferred to a prepared 96-well plate planted with SRBC, gently pipetted to ensure complete resuspension, and incubated at 4 °C for 15 min. After incubation, they were centrifuged at 600× *g* for 5 min at 4 °C, the supernatants were discarded, and 200 μL of glycine-HCl sample incubation solution with the same concentration as during incubation was added per well. They were gently mixed by resuspending and centrifuged again. The above steps were repeated twice to wash away excess antibodies that could not bind to the SRBC antigens due to the acidic environment.

The SRBCs were then resuspended to 100 μL per well normal saline (0.9% NaCl) with 1 μg/mL rabbit anti-mouse IgG (H + L) AF488 antibody and incubated at 4 °C in the dark for 20 min. Following incubation, the SRBCs were washed with normal saline by the above-mentioned centrifugation procedure twice before resuspended in normal saline for 200 μL per well, and the detection was performed using a flow cytometer (BD LSRFortessa TM X-20). After detection, a curve with the antibody fluorescence intensity correlating to glycine concentration was generated, where the negative reciprocal of the slope of the linear fit line between antibody fluorescence intensity and glycine concentration was taken as the relative affinity value (RAV).

### 4.6. Statistical Analysis

Statistical analyses were performed using Graphpad Prism 9. The data of the WT mice samples from different colleting time points (Figure 4) were analyzed with one-way ANOVA. The data of WT mice and ShipΔB mice samples from different colleting timepoints (Figure 5) were analyzed with an unpaired *t*-test per time point. The data are represented as mean ± SEM. A *p*-value < 0.05 was considered significant in the statistical analyses. See Figure 4 and Figure 5 for more details.

## 5. Patents

A patent application based on the study has been submitted (Chinese patent application number: 202510110156.3), and H.Z., C.S., F.L., Y.J., S.L. and Y.Z. are listed as inventors.

## Data Availability

Any additional information required to reanalyze the data in this study can be requested by contacting the lead contact (H.Z.).

## Supplementary Materials

The following supporting information can be downloaded at: https://www.mdpi.com/article/10.3390/ijms26083664/s1.
