# Supplementary material for: Flow Cytometry-Based Rapid Assay for Antigen Specific Antibody Relative Affinity in SRBC-Immunized Mouse Models
Source: Int J Mol Sci. 2025 Apr 12;26(8):3664. doi: 10.3390/ijms26083664 (PMC12027684; doi:10.3390/ijms26083664)
Supplement: Supplementary file 1 [file ijms-26-03664-s001.zip › ijms-3511276-supplementary.pdf]

**Supplementary Material**

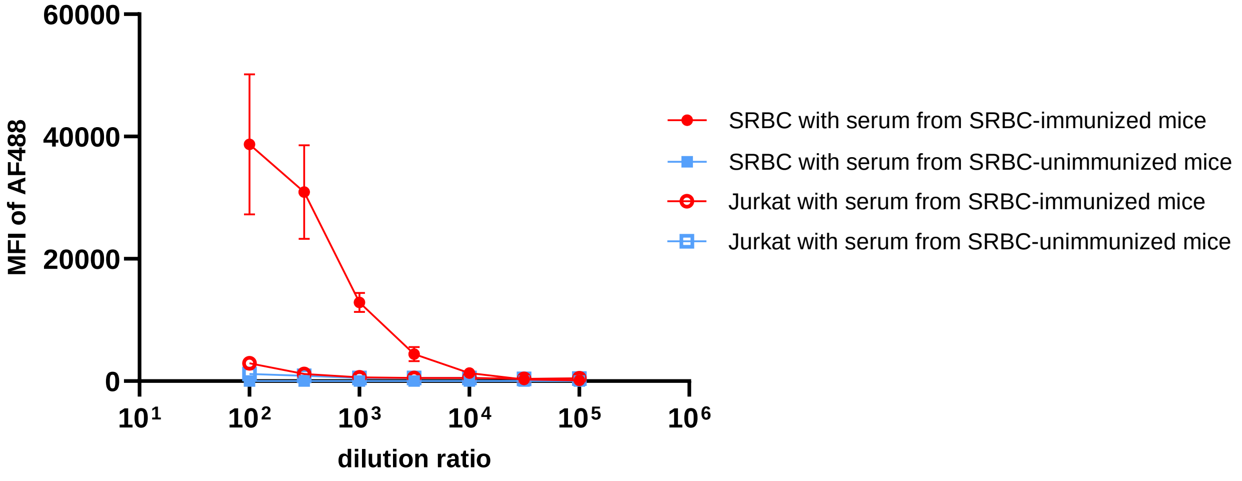


**Figure S1.** The titration curves of SRBC and Jurkat cells incubated with serum samples from wild-type mice on Day 35 of SRBC immunization or from unimmunized mice, respectively. The data are represented as mean ± SEM (N=3).

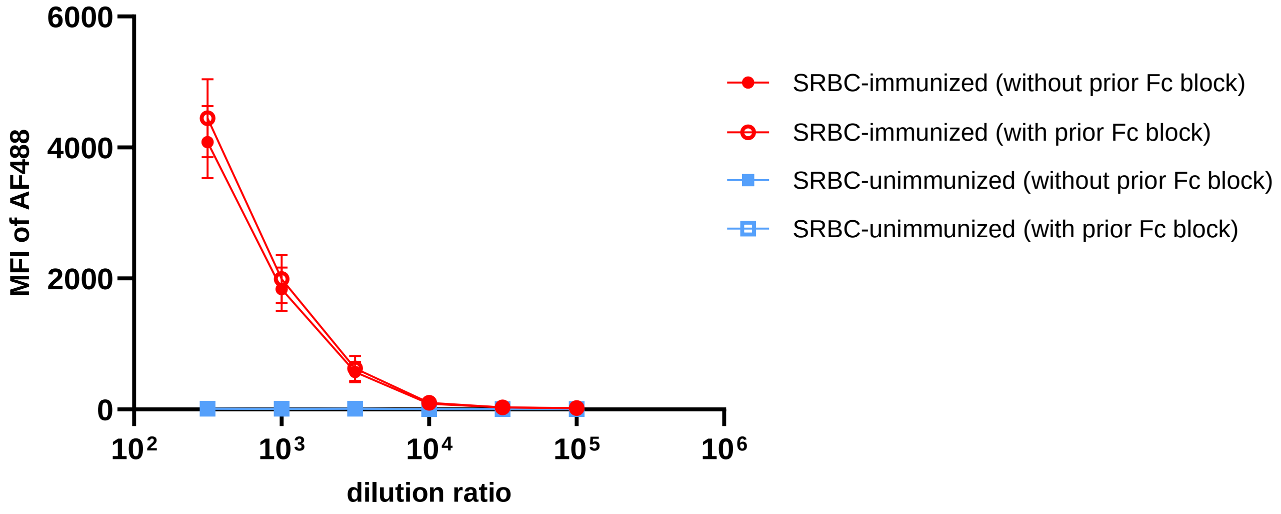


**Figure S2.** The titration curves of SRBC cells incubated with serum samples from wild-type mice on Day 35 of SRBC immunization or from unimmunized mice, with or without prior Fc block, respectively. The data are represented as mean ± SEM (N=3).

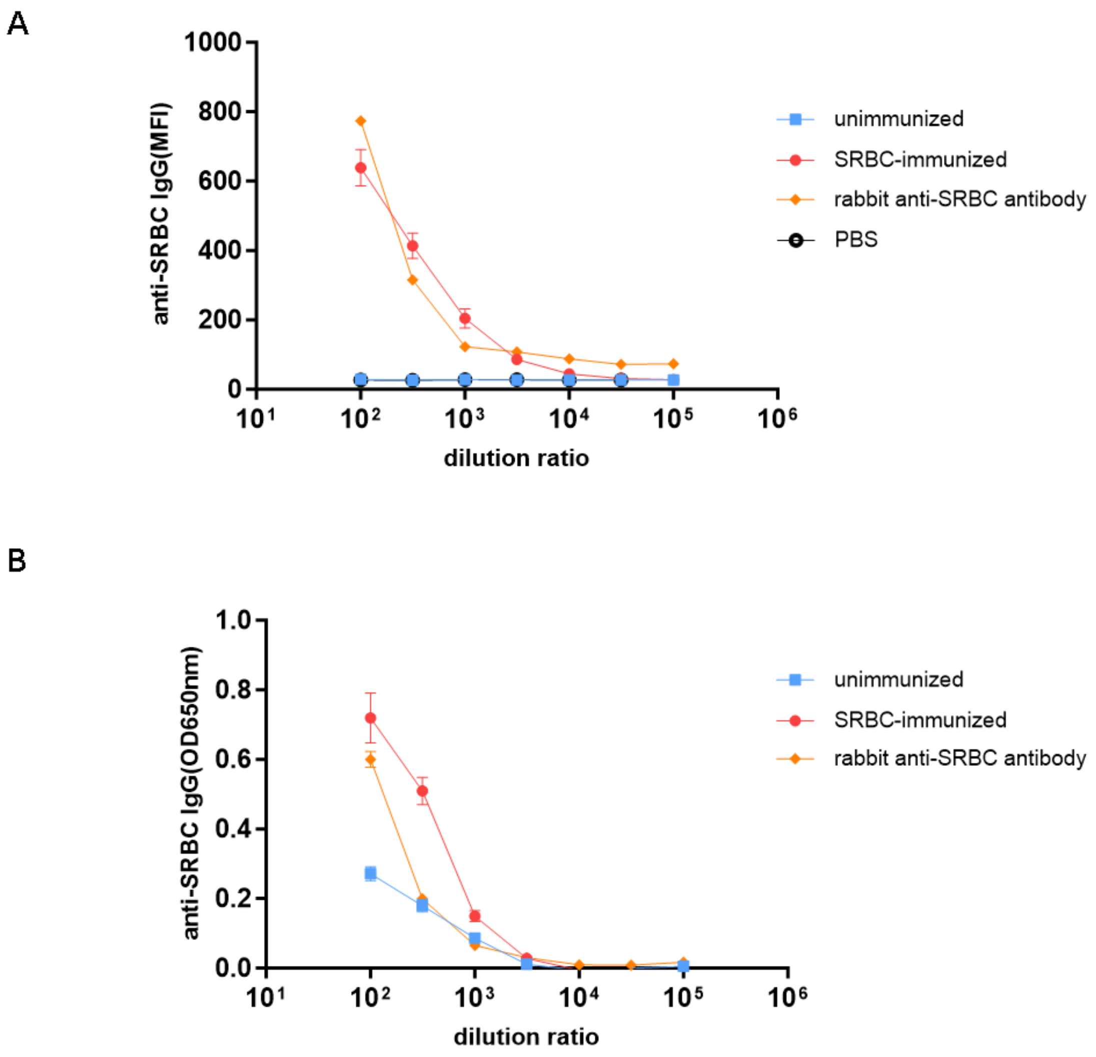


**Figure S3.** The titration curves of commercialized rabbit anti-SRBC antibody or serum samples from wild-type mice on Day 14 of SRBC immunization or from unimmunized mice incubated with SRBC cells (A) or SRBC antigens coated on ELISA plates (B), respectively. The titration curves were determined with flow cytometry (A) or ELISA (B), respectively. The data are represented as mean ± SEM (N=3).

## Supplementary Methods

### Detection of serum antibody binding to SRBC and Jurkat cells

The total required amount of SRBC cells following the standard of 1*10^6 SRBCs per well of a 96-well plate was added into a centrifuge tube and washed with PBS by centrifugation at 600 xg for 5 minutes at 4 °C twice before being resuspended to an appropriate concentration in PBS. Then, the Jurkat cells were added into centrifuge tube with the required amount of 2*10^5 cells per well of a 96-well plate, washed with PBS by centrifugation at 400 xg for 5 minutes at 4°C, and removed supernatant twice. The cells were both added to a 96-well U-bottom plate with the required amount in 100 µl per well, respectively. The serum samples were diluted in a 3.16-fold gradient series, added to the 96-well plate planted with cells, mixed well, and incubated at 4°C for 20 minutes. All wells were then washed three times with PBS by the above-mentioned centrifugation procedures, respectively, before being resuspended to 50 µl FACS buffer (PBS containing 1 mM ethylene diamine tetra acetic acid (EDTA), 2% Fetal Bovine Serum (FBS) (ExCell Bio, Cat#FSP500) with 1 µg/ml Alexa Fluor ™ 488 rabbit anti-mouse IgG(H+L) antibody (Invitrogen, Cat#A-11059), and incubated at 4°C in the dark for 20 minutes. Following incubation, the cells were washed with PBS by the above-mentioned centrifugation procedures twice, respectively, before being resuspended in FACS buffer for 200 µl per well, and the detection was performed with flow cytometer (BD FACSymphony), generating the titration curves of the test samples with Graphpad prism.

### Detection of serum antibody binding to SRBC with or without prior Fc block

The total required amount of SRBC cells were followed standard of 2*10^7 SRBCs per well, and the washing and adding procedures were followed as mentioned above. The SRBC cells were divided into two groups, and one was resuspended with 50ul FACS buffer, while the other was resuspended with 50 µl 1:500 2.4G2 (Bio×Cell, Cat# BE0307) stain solution (in PBS); both two were placed at 4°C for 20 minutes. All wells were then washed twice with PBS by centrifugation at 600 xg for 5 minutes at 4°C, and the supernatant was removed. Then, the incubation of serum samples and Alexa Fluor ™ 488 rabbit anti-mouse IgG(H+L) antibody were followed as mentioned above after being washed twice and resuspended. Then, the samples were detected by flow cytometer (BD FACSymphony), generating the titration curves of the test samples by Graphpad prism.

### ELISA

The enzyme-linked immunosorbent assay (ELISA) for anti-SRBC antibodies was performed following a standard protocol. To prepare SRBC antigen, SRBC cells were sonicated in RIPA Lysis Buffer (Beyotime, Cat#P0013B). The condition of sonication included four cycles of 10 seconds of sonication and 5 seconds of resting, both on ice. The lysate was centrifuged at 16000 xg at 4℃ for 5 minutes, and the supernatant was obtained as antigen. The 96-well

plates we used were coated with 5 μg/mL lysate at 50 μl/well and incubated overnight at 4 °C. The plates were then washed three times with 200 μl/well PBST (0.05% Tween-20 in PBS) and blocked with 200 μl/well 5% BSA solution (in PBS) for at least 1 hours at room temperature. After blocking, the plates were washed by PBST for three times, and 3.16 serial dilutions of serum samples from mice and commercialized rabbit anti-SRBC antibody (Yuanye, Cat#S25861) were added to the wells and incubated for 1 hour at room temperature, followed by three PBST washes. Among the samples, serum samples of unimmunized mice were used as negative control, while PBS alone was used as blank control. The wells received serum samples, and PBS in plates were added to a 1:5000 PBS solution of HRP goat anti-mouse IgG antibody (Jackson ImmunoResearch, Cat#115-035-071), while the commercialized rabbit anti-SRBC antibody was added to a 1:5000 PBS solution of HRP goat anti-rabbit IgG antibody (UElandy, Cat#H6162S), with 50 μl added to each well. Then, the plates were incubated for 1 hour and washed five times, followed by TMB substrate reaction for 30 minutes. Absorbance was measured at 650 nm using a microplate reader.
